# From dreams to parkinsonism: tracking the journey

**DOI:** 10.1093/brain/awz155

**Published:** 2019-06-26

**Authors:** Michele T M Hu

**Affiliations:** Oxford, UK

## Abstract

This scientific commentary refers to ‘Evolution of prodromal Parkinson’s disease and dementia with Lewy bodies: a prospective study’ by Fereshtehnejad *et al.* (doi:10.1093/brain/awz111)

This scientific commentary refers to ‘Evolution of prodromal Parkinson’s disease and dementia with Lewy bodies: a prospective study’ by Fereshtehnejad *et al.* (doi:10.1093/brain/awz111)

Perhaps ‘the’ primary health challenge of the 21st century for the developed world is to find cures for neurodegenerative disorders including Alzheimer’s disease and Parkinson’s disease. The number of people living with Parkinson’s disease globally is projected to double from 2015 to 2040, hence if Parkinson’s disease were an infectious condition it would rightly be called a pandemic (Dorsey and Bloem, 2018). Arguably, the success of vaccination programmes introduced to the developed world in the past century is one of the factors that has led to this neurodegenerative pandemic, by increasing life expectancy and thus the incidence of ageing-related disorders. But immunomodulatory approaches are also relevant in the search for a cure, with α-synuclein vaccination and monoclonal antibody therapies now very real possibilities for clinical trials. Hence, vaccines may paradoxically help to both create and cure a future neurodegenerative pandemic.

Over the past 30 years, billions of dollars have been invested by the pharmaceutical industry in the search for a cure for Parkinson’s disease, with 16 compounds all testing negative in completed large-scale trials (Espay *et al.*, 2017). These studies focused on patients with an established diagnosis of Parkinson’s disease, based on the presence of clinical motor features of bradykinesia, tremor and rigidity. The studies may have failed in part because intervention was attempted too late in the neurodegenerative cascade. Focus over the past decade has therefore shifted increasingly to earlier disease stages, with calls for a new research framework encompassing clinical features, pathological findings, genetics and molecular mechanisms to re-define Parkinson’s disease (Berg *et al.*, 2013). However, one of the greatest challenges is that motor symptoms meeting the clinical criteria for diagnosis generally only become apparent when dopaminergic cell loss in the contralateral brainstem (substantia nigra pars compacta) approaches 40–50%, with dopamine depletion of ∼60–70% (Berg *et al.*, 2013).

Increasing recognition that non-motor features including olfactory dysfunction, REM sleep behaviour disorder (RBD), autonomic dysfunction (constipation and urinary dysfunction), visual dysfunction and anxiety/depression can predate the onset of motoric features by up to 20 years has led to renewed interest in the concept of ‘prodromal’ Parkinson’s disease. From the Greek *prodromos*, meaning ‘running before’, prodromal Parkinson’s disease represents a much earlier disease phase for intervention, with higher densities of salvageable neurons. It would also allow intervention without confounding placebo or nocebo effects of symptomatic therapies, including levodopa—a highly effective rescue treatment for motor symptoms that may be neuroprotective in its own right.

In this issue of *Brain*, Fereshtehnejad and co-workers focus on one particular group of prodromal patients, namely those with RBD, who might just represent a well-stratified and delineated group suitable for early intervention. Therapies applied to prodromal groups could have a real chance of curing Parkinson’s disease and preventing the onset of motor symptoms. Even if a cure is not possible, slowing the relentless trajectory of deterioration would also be a significant game changer. REM sleep is the phase of sleep in which dreaming occurs, and is characterized by rapid, desynchronized EEG activity, loss of postural muscle tone, muscle twitching and episodic bursts of rapid eye movement. REM sleep is regulated by sleep centres in the pontine brainstem, but its function remains unknown. RBD occurs when the descending neuronal glutamatergic projections that normally induce muscle atonia are disrupted. It may be idiopathic or secondary, with the latter resulting from pathology of the pontine subcoeruleus/magnocellularis nuclei and their projections, for example due to ischaemia, demyelination or a tumour.

Clinically, RBD is characterized by dream-enacting behaviours and nightmares in association with REM sleep without muscle atonia. Secondary RBD is seen in patients with disorders including Parkinson’s disease (25–58%), dementia with Lewy bodies (DLB) (70–80%), and multiple system atrophy (MSA) (90–100%). Some medications (e.g. antidepressants and β-adrenergic blockers) can also trigger RBD. Although RBD is a relatively new kid on the block, having first been described just over 30 years ago (Schenck *et al.*, 1986), evidence from a growing number of well-phenotyped RBD cohorts worldwide has shown that >80% of those affected will eventually develop an α-synuclein disorder, of which Parkinson’s disease is the most common, followed by dementia (principally DLB but also other clinically non-classifiable forms) and MSA. The largest ever study of 1280 polysomnographically-diagnosed idiopathic RBD subjects from 24 International RBD Study Group sleep centres by a single author group, found an overall conversion rate from idiopathic RBD to an overt neurodegenerative syndrome of 6.3% per year (Postuma *et al.*, 2019).

The study by Fereshtehnejad *et al.* (2019) is the first of its kind to fully map the process of conversion from prodromal to motor Parkinson’s disease using an unbiased and comprehensive longitudinal phenotyping approach (average 5-year follow-up) to address key questions on how and when specific non-motor features manifest*.* Between 2004 and 2016, the authors recruited and then annually followed up 154 polysomnographically-proven idiopathic RBD individuals, of whom 55 phenoconverted to defined parkinsonism or dementia. Longitudinal data on multiple prodromal features including the Unified Parkinson’s Disease Rating Scale (UPDRS) parts I–III, quantitative motor tests, olfaction, colour vision, cognition and autonomic functions were gathered annually. Importantly, the same measures were also assessed in 102 age- and sex-matched healthy control subjects to reveal the effects of normal ageing on these non-motor features. By looking backwards from the time of a dementia/parkinsonism diagnosis, the authors were able to examine trajectories of each prodromal non-motor and motor feature using mixed effect models.

As can be seen in Fig. 1, which maps the progression of non-motor manifestations from prodromal stages to phenoconversion, olfactory loss was the first to develop with predicted onset >20 years before phenoconversion. This was followed by impaired colour vision, constipation and erectile dysfunction starting 10–16 years prior to conversion. Slight urinary dysfunction and subtle cognitive decline manifest 7–9 years before conversion.

**Figure 1:**
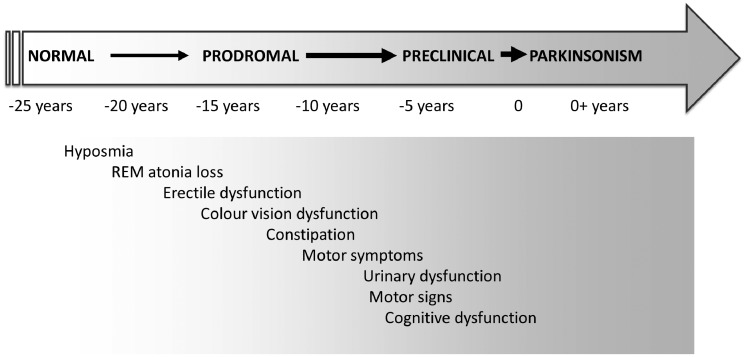
**Time-line of non-motor manifestations from prodromal stages to phenoconversion**.

Mild motor symptoms including reported alterations in handwriting, turning in bed, walking, salivation and speech, and reduced facial expression manifested 7–11 years prior to Parkinson’s disease diagnosis, and gradually increased up to the point of diagnosis. This was also mirrored in physician-rated scales including the UPDRS, and semiquantitative motor testing including the Purdue Pegboard test and the Timed Up and Go test (see Fig. 1 in Fereshtehnejad *et al.*, 2019). In terms of cardinal motor feature progression, bradykinesia followed by rigidity and tremor incrementally increased over time to diagnosis (see Fig. 2 in Fereshtehnejad *et al.*, 2019). Motor and cognitive abnormalities emerged relatively late in the phenoconversion process, and had reached only 20–30% maximal values at the time of phenoconversion. Most manifestations progressed in a linear fashion, except motor symptoms and signs, which exhibited a more rapid progression during the last 2 years prior to phenoconversion, consistent with many other studies in early Parkinson’s disease. With regards to diagnostic potential, hyposmia or olfactory dysfunction was the most specific feature to predict phenoconversion. The authors were able to demonstrate that the evolution of prodromal manifestations was similar to that predicted by pathological staging models of Parkinson’s disease (including the Braak model).

This study is not without some important limitations and caveats. Estimates become increasingly imprecise at the longer time intervals because many patients develop disease in the first few years after baseline. Hence the further from the time of phenoconversion, the smaller the sample size. Weighted regression and mixed effect models were applied in an attempt to mitigate this, however validation in other cohorts is needed. This study relied on clinical manifestations alone, with the associated limitations of rating scales, and lacked digital high frequency measures that could estimate motor dysfunction in a truly quantitative manner, approaches now being trialled with reasonable success by others in Parkinson’s disease/RBD (Arora *et al.*, 2018). There are currently no nuclear imaging biomarkers for Parkinson’s disease that directly estimate the pathological burden of α-synuclein, although efforts to produce suitable ligands are ongoing. Tracers that could reveal the trajectory of dopaminergic cell loss are currently being assessed longitudinally in RBD, in addition to promising MRI markers (see Barber *et al.*, 2017 for review).

Debates about the generalizability of RBD as a prodromal form of Parkinson’s disease continue to rage, with some suggesting that prodromal cohorts should instead be recruited from those with rare monogenic forms of Parkinson’s disease (estimated to represent <10% of community-acquired cases) or from elderly normal populations stratified by the presence of non-motor symptoms. This latter approach, however, may simply not generate a sufficient number of Parkinson’s disease convertors for future trials. Trials are likely to be limited to 1–2 years owing to funding constraints, with phenoconversion rates or surrogate imaging/other biomarkers as primary study end-points. Many of the arguments querying the generalizability of sleep cohort-ascertained RBD patients to Parkinson’s disease stem fromthe high male to female ratios (8:1) seen in RBD, different from the 2:1 male to female ratio in Parkinson’s disease. However, recent population-based studies using gold-standard polysomnography for RBD diagnosis have found crude estimated RBD prevalences of 1–2% in the over 60s with only a slight gender bias, approaching that of Parkinson’s disease (Kang *et al.*, 2013; Haba-Rubio *et al.*, 2018). Although limited numbers of RBD subjects have undergone post-mortem examination, findings so far point to an α-synuclein disorder pathologically similar to Parkinson’s disease, with numerous cortical/subcortical Lewy bodies. Those of us who have followed-up sizeable cohorts of RBD subjects and seen first-hand the relentless phenoconversion rates, would therefore argue that the time is now ripe for neuroprotective trials to focus on this promising prodromal group.

## Funding

M.T.M.H is employed by Oxford University and Oxford University Hospitals NHS Foundation Trust, with research funded by the Monument Trust Discovery Award from Parkinson’s UK and the National Institute for Health Research (NIHR) Oxford Biomedical Research Centre based at Oxford University Hospitals NHS Trust and University of Oxford.

## Competing interests

The author reports no competing interests.
